# Abnormal corneal nerve morphology and brain volume in patients with schizophrenia

**DOI:** 10.1038/s41598-022-05609-w

**Published:** 2022-02-03

**Authors:** Georgios Ponirakis, Reem Ghandi, Amani Ahmed, Hoda Gad, Ioannis N. Petropoulos, Adnan Khan, Ahmed Elsotouhy, Surjith Vattoth, Mahmoud K. M. Alshawwaf, Mohamed Adil Shah Khoodoruth, Marwan Ramadan, Anjushri Bhagat, James Currie, Ziyad Mahfoud, Hanadi Al Hamad, Ahmed Own, Peter M. Haddad, Majid Alabdulla, Rayaz A. Malik, Peter W. Woodruff

**Affiliations:** 1 Department of Medicine, Weill Cornell Medicine-Qatar, Qatar Foundation, Education City, Doha, Qatar; 2 Psychiatry Hospital, Mental Health Service, Hamad Medical Corporation, Doha, Qatar; 3 Neuroradiology, Hamad General Hospital, Hamad Medical Corporation, Doha, Qatar; 4 Radiology, University of Arkansas for Medical Sciences, Little Rock, AR USA; 5 Geriatric, Rumailah Hospital, Hamad Medical Corporation, Doha, Qatar; 6 College of Medicine, Qatar University, Doha, Qatar; 7 Institute of Cardiovascular Science, University of Manchester, Manchester, UK; 8 Division of Psychology and Mental Health, School of Health Sciences, University of Manchester, Manchester, UK; 9 Department of Neuroscience, School of Medicine,, University of Sheffield, Western Bank, Sheffield, S10 2TN South Yorkshire UK

**Keywords:** Diagnostic markers, Neurological disorders, Endocrine system and metabolic diseases

## Abstract

Neurodevelopmental and neurodegenerative pathology occur in Schizophrenia. This study compared the utility of corneal confocal microscopy (CCM), an ophthalmic imaging technique with MRI brain volumetry in quantifying neuronal pathology and its relationship to cognitive dysfunction and symptom severity in schizophrenia. Thirty-six subjects with schizophrenia and 26 controls underwent assessment of cognitive function, symptom severity, CCM and MRI brain volumetry. Subjects with schizophrenia had lower cognitive function (P ≤ 0.01), corneal nerve fiber density (CNFD), length (CNFL), branch density (CNBD), CNBD:CNFD ratio (P < 0.0001) and cingulate gyrus volume (P < 0.05) but comparable volume of whole brain (P = 0.61), cortical gray matter (P = 0.99), ventricle (P = 0.47), hippocampus (P = 0.10) and amygdala (P = 0.68). Corneal nerve measures and cingulate gyrus volume showed no association with symptom severity (P = 0.35–0.86 and P = 0.50) or cognitive function (P = 0.35–0.86 and P = 0.49). Corneal nerve measures were not associated with metabolic syndrome (P = 0.61–0.64) or diabetes (P = 0.057–0.54). The area under the ROC curve distinguishing subjects with schizophrenia from controls was 88% for CNFL, 84% for CNBD and CNBD:CNFD ratio, 79% for CNFD and 73% for the cingulate gyrus volume. This study has identified a reduction in corneal nerve fibers and cingulate gyrus volume in schizophrenia, but no association with symptom severity or cognitive dysfunction. Corneal nerve loss identified using CCM may act as a rapid non-invasive surrogate marker of neurodegeneration in patients with schizophrenia.

## Introduction

Schizophrenia is a major psychosis associated with significant neurocognitive dysfunction and disability. Both neurodevelopmental and neurodegenerative processes may contribute to schizophrenia^1,2^. Currently, there are no validated surrogate markers of neuronal pathology in schizophrenia.

In patients with schizophrenia, cross-sectional MRI studies have reported a lower brain volume^3,4^ and longitudinal studies have shown a progressive reduction in brain volume^5^. However, both global and regional alterations in brain structure have not been associated with the presence or severity of psychosis^6^. Therefore, brain MRI has not been utilized as a clinical-decision making tool in schizophrenia^7^. Additional studies utilizing electroretinography (ERG) and optical coherence tomography (OCT) have shown a reduction in photoreceptor and bipolar cell activity and retinal nerve fiber layer and macular volumes, respectively^8^, suggesting more widespread neurodegeneration in schizophrenia. Atrophy of ganglion cell axons could be attributed to dopamine dysregulation^9^. However, it is important to note that in schizophrenia; smoking, poor nutritional intake with vitamin B_12_ and D deficiency as well as physical inactivity and antipsychotic medications can lead to obesity, hypertension, metabolic syndrome and diabetes which may also contribute to neurodegeneration^10–12^.

Corneal nerves in the sub-basal plexus are comprised of unmyelinated C-fibers that convey thermal and mechanical sensation to protect the eye from injury^13^. Corneal confocal microscopy (CCM) is a rapid non-invasive ophthalmic imaging technique that has been utilized to demonstrate corneal nerve loss in a range of peripheral neuropathies including diabetic neuropathy^14^, HIV neuropathy^15^, and recently long-COVID^16^. We and others have also shown corneal nerve fiber loss in patients with dementia^17^ and multiple sclerosis^18^.

It is hypothesized that patients with schizophrenia will have evidence of corneal nerve loss independent of other risk factors for neuropathy, including metabolic syndrome and diabetes. This is the first study to quantify corneal nerve pathology and regional MRI brain volumes in relation to symptom severity and cognitive function in patients with schizophrenia.

## Methods

### Study design

This is an exploratory cross-sectional study. Subjects with schizophrenia were enrolled from the Psychiatry Hospital in Qatar and healthy controls were enrolled from Weill Cornell Medicine-Qatar, Rumailah Hospital and the Psychiatry Hospital in Qatar between August 26, 2019 and March 11, 2020. The authors assert that all procedures contributing to this work comply with the ethical standards of the relevant national and institutional committees and with the Helsinki Declaration. All procedures involving human subjects/patients were approved by the Weill Cornell Medicine in Qatar IRB (# 19-00031) and Hamad Medical Corporation IRB (# IRGC-04-SI-17-166). Written informed consent was obtained from all subjects/patients.

### Inclusion and exclusion criteria

Subjects with schizophrenia and healthy controls aged 18–65 years old were recruited. Exclusion criteria for schizophrenia included mental illness other than schizophrenia or causes of peripheral neuropathy other than diabetes. Exclusion criteria for controls included mental illness, causes of peripheral neuropathy or diabetes. Other exclusion criteria included substance abuse except nicotine and caffeine, corneal trauma, dystrophy, surgery, severe dry eyes or allergy to local anesthetics.

### Diagnosis

The diagnosis of schizophrenia was made by psychiatrists using the Diagnostic and Statistical Manual of Mental Disorders (DSM-5) criteria^19^. Each person had two or more core symptoms, one of which was hallucinations, delusions, or disorganized speech for at least one month and gross disorganization and diminished emotional expression. Other DSM-5 criteria for the diagnosis of schizophrenia included (1) level of work, interpersonal relations or self-care significantly below the start of symptoms, (2) signs of disturbance for at least 6 months, (3) schizoaffective disorder and depressive or bipolar disorder with psychotic symptoms ruled out and (4) the disturbance was not caused by substance abuse or another medical condition. Subjects with schizophrenia underwent clinical interview and psychiatric assessment using the Positive and Negative Syndrome Scale (PANSS) English and Arabic version^20^ and cognitive function assessment using the Montreal Cognitive Assessment (MoCA) English and Arabic version test, whereas healthy controls underwent MoCA only. Both the PANSS and MoCA were administered by one investigator who was an English and Arabic speaker and was trained on the use and interpretation of the PANSS and MoCA. The MoCA was utilized as a continuous variable to assess cognitive function but not to define cognitive impairment in patients with schizophrenia. It assesses a broad range of cognitive domains, including memory, executive functions, attention and visuospatial ability, has a short administration time and widespread international use^21^.

### Demographic and clinical characteristics

Age, sex, duration of schizophrenia, presence of diabetes, blood pressure, body mass index (BMI), medical history, HbA1c, lipid profile, creatinine, vitamin B_12_ and vitamin D were recorded. Hypertension was defined according to the WHO/ISH Guidelines^22^. Hyperlipidemia was defined according to a total cholesterol level ≥ 6.2 mmol/L and/or triglyceride level of ≥ 2.3 mmol/L or if the patient was treated with a statin. Obesity was classified according to WHO criteria with a BMI ≥ 30 kg/m^2^^23^. Current smoking cigarette was defined as having smoked at least one cigarette every day for ≥ 1 year. Metabolic syndrome was defined using the WHO 1999 criteria^24^.

### Corneal confocal microscopy (CCM) image acquisition and analysis

CCM was performed with the Heidelberg Retinal Tomograph III Rostock Cornea Module (Heidelberg Engineering GmbH, Heidelberg, Germany). A 63× objective lens with a numerical aperture of 0.9 and a working distance, relative to the applanating cap (TomoCap, Heidelberg Engineering GmbH, Heidelberg, Germany) of 0.0–3.0 mm was used. The images produced using this lens are 400 μm × 400 μm with a 15° × 15° field of view and 10 μm/pixel transverse optical resolution. The cornea was locally anesthetized by instilling 1 drop of 0.4% benoxinate hydrochloride (Chauvin Pharmaceuticals, Chefaro, UK) and Viscotears (Carbomer 980, 0.2%, Novartis, UK) was used as the coupling agent between the cornea and the TomoCap and between the TomoCap and objective lens. Images of the sub-basal nerve plexus were captured using the “section” mode. CCM image extraction was performed at a separate time by an investigator who was blinded to the patient diagnosis. Three to five representative images of the sub-basal nerve plexus were selected per eye. CCMetrics, a validated image analysis software^25^ was used to quantify corneal nerve fiber density (CNFD, fibers/mm^2^), branch density (CNBD, branches/mm^2^), fiber length (CNFL, mm/mm^2^) and CNBD:CNFD ratio.

### MRI brain acquisition and volume analysis

MRI was performed on a superconductive magnet operated at 3 T (Skyra, Siemens). A T1-weighted 3D magnetization prepared rapid acquisition gradient echo sequence (MPRAGE) was obtained in the sagittal plane with a 1 mm slice thickness, repetition time of 1900 ms, echo time of 2.67 ms and 2.46 ms, inversion time of 1100 ms and 900 ms, flip angle of 9° and 15°, and FOV = 240 × 100. Coronal and axial reformatted MPRAGE images were reconstructed from the sagittal 3D sequence. MRI T1-weighted 3D MPRAGE sequences were processed using NeuroQuant (NQ), an FDA approved fully automated software^26^ to segment brain structures and measure the intracranial volume (ICV) adjusted percentage of the whole brain, cortical gray matter, ventricle, cingulate gyrus, hippocampi, amygdala, thalamus and entorhinal cortex.

### Peripheral neuropathy assessments

Vibration perception threshold (VPT) was measured as volts (V) on the pulp of the large toe using a Neurothesiometer (Horwell, Scientific Laboratory Supplies, Wilford, Nottingham, UK). Sudoscan, was used to measure sudomotor function in the feet expressed as electrochemical skin conductance (ESC) and measured in microSiemens (µS).

### Statistical analysis

This was the first exploratory study of CCM in schizophrenia, therefore no power calculation was determined. Continuous and categorical variables between subjects with schizophrenia and healthy controls were compared using the unpaired t-test and chi-square, respectively. Pearson's correlation coefficients were calculated to determine the association between intracranial volume of the cingulate gyrus and corneal nerve branch to fiber density ratio.

Univariate analysis by simple linear regression was performed between independent variables and corneal nerve measures and MRI brain volume as the dependent variable. The multiple linear regression analysis included all variables with P ≤ 0.05 at the bivariate level. The regression coefficient (beta) and the corresponding 95% confidence intervals (95% CI) are presented. The R-squared values are presented. The model that yielded the highest R-squared value was the preferred one.

Receiver operating characteristic (ROC) curve analysis was used to determine the diagnostic ability of CCM, MRI brain volume and cognitive function to distinguish subjects with schizophrenia from healthy controls. The area under curve (AUC), and cut-off points with the maximal sum of sensitivity and specificity were selected.

Analysis of all data was performed using Statistical Package for the Social Sciences (SPSS). A two-tailed P value of ≤ 0.05 was considered significant.

## Results

### Demographic and clinical characteristics

Subjects (n = 36) with a mean duration of 11.1 ± 8.1 years of schizophrenia and healthy controls (n = 26) were studied. The clinical characteristics of these groups are summarized in Supplementary Table 1. Both groups had comparable age (33.7 ± 11.1 years vs 35.3 ± 11.1 years, t = 0.5, P = 0.59), sex (P = 0.38), percentage of cigarette smokers (27.8% vs 15.4%, χ2 = 1.3, P = 0.25), percentage with obesity (P = 0.21), systolic and diastolic blood pressure (P = 0.98), HbA1c (P = 0.06), creatinine (P = 0.80), total cholesterol (P = 0.32), triglycerides (P = 0.88), HDL (P = 0.64), LDL (P = 0.39), 25OHD (P = 0.47) and vitamin B_12_ (P = 0.39). However, subjects with schizophrenia had a higher BMI (32.9 ± 9.1 kg/m^2^ vs 27.5 ± 5.2 kg/m^2^, t = 2.9, P < 0.001), percentage with hypertension (44.4% vs 11.5%, χ2=7.7, P < 0.01), and hyperlipidemia (33.3% vs 7.7%, χ2 = 5.7, P < 0.05) compared to healthy controls. The prevalence of diabetes and metabolic syndrome in patients with schizophrenia was 27.8% (n = 10/36) and 30.6% (n = 11/36), respectively. The HbA1c of subjects with schizophrenia and diabetes was 6.6 ± 1.6%. The percentage of antipsychotic drug use which was more likely to cause dry eyes (1–10% risk of dry eyes) was lower than those with a 0.1–1% risk of dry eyes (31.7% vs 68.3%).

PANSS total symptom severity ranged from 41 to 100 with a mean of 68.4 ± 12.7. Cognitive function measured by MoCA was significantly lower in subjects with schizophrenia compared to healthy controls (MoCA: 24.2 ± 3.6 vs 26.8 ± 2.8, t = 3.0, P ≤ 0.01). After adjusting for creatinine, the association of schizophrenia with reduced cognitive function remained significant (β coefficient: − 2.6, 95% CI − 4.7, − 0.6, P = 0.01). The association between schizophrenia and cognitive function also remained significant after adjusting for diabetes, hypertension, hyperlipidemia and BMI, although the adjusted r^2^ was lower after adjusting for these imbalances (r^2^: 0.19 vs 0.23).

### Peripheral neuropathy assessment (Table 1)

**Table 1:** Comparison of cognitive function, corneal nerve morphology, volumetric brain MRI and measures of peripheral neuropathy between subjects with schizophrenia and controls.

	Controls (n = 26)	Schizophrenia (n = 36)	P value	T value
MoCA score	26.8 ± 2.8	24.2 ± 3.6	< 0.01	3.0
**Corneal nerve morphology**				
CNFD (fibers/mm^2^)	37.3 ± 6.7	27.9 ± 9.1	< 0.0001	4.6
CNBD (branches/mm^2^)	87.6 ± 29.3	41.9 ± 28.9	< 0.0001	6.0
CNFL (mm/mm^2^)	24.6 ± 3.9	16.7 ± 5.4	< 0.0001	6.5
CNBD:CNFD ratio	2.4 ± 0.8	1.4 ± 0.7	< 0.0001	4.7
**Volumetric analysis of brain MRI**				
Whole brain (ICV %)	76.5 ± 3.8	77.2 ± 2.3	0.61	–0.5
Cortical gray matter (ICV %)	34.2 ± 3.4	34.2 ± 1.7	0.99	–0.001
Ventricle (ICV %)	1.4 ± 0.8	1.6 ± 0.6	0.47	–0.7
Cingulate gyrus (ICV %)	0.89 ± 0.08	0.83 ± 0.07	< 0.05	2.2
Hippocampus (ICV %)	0.54 ± 0.03	0.51 ± 0.05	0.10	1.7
Amygdala (ICV %)	0.24 ± 0.03	0.23 ± 0.03	0.68	0.4
Thalamus (ICV %)	0.92 ± 0.09	0.97 ± 0.08	0.20	–1.3
Entorhinal cortex (ICV %)	0.33 ± 0.05	0.35 ± 0.06	0.26	–1.2
**Measures of peripheral neuropathy**				
VPT (V)	4.7 ± 2.9	5.9 ± 5.4	0.32	–1.0
ESC feet (µS)	70.1 ± 12.7	62.9 ± 19.9	0.09	1.7

Numeric variables and frequency distribution for categorical variables are summarized as mean ± standard deviation or n (%). Variables were compared using unpaired t-test. Categorical variables were compared using x^2^.

*MoCA* montreal cognitive assessment, *CNFD* corneal nerve fiber density, *CNFL* length, *CNBD* branch density, *VPT* vibration perception threshold, *ESC* electrochemical skin conductance, *ICV* intra cranial volume.

Subjects with schizophrenia had comparable vibration perception threshold (VPT) (P = 0.32) and sudomotor function (P = 0.09) to control subjects.

### Corneal nerve fiber measures (Tables 1, 2 and Fig. 1)

**Table 2:** The association of cognitive function, corneal nerve fiber measures, cingulate gyrus volume with schizophrenia after adjustment for cofounders.

	Adjusted mean difference	95% confidence interval	P value
**CNFD (fibers/mm** ^**2**^ **) (min.–max: 10.4–49.1)**			
Schizophrenia	− 5.2	− 9.4, − 1.0	< 0.05
Smoking cigarette	− 6.5	− 11.2, − 1.8	< 0.01
BMI (kg/m^2^)	− 0.4	− 0.6, − 0.1	0.01
VPT (V)	− 0.6	− 1.0, − 0.1	0.01
**CNBD (branches/mm** ^**2**^ **) (min.–max: 7.6–142.2)**			
Schizophrenia	− 41.4	− 62.0, − 20.8	< 0.0001
BMI (kg/m^2^)	− 0.3	− 1.3, 0.8	0.63
Sleep (h)	− 3.0	− 6.4, 0.4	0.08
**CNFL (mm/mm** ^**2**^ **) (min.–max: 5.9–32.9)**			
Schizophrenia	− 7.1	− 10.6, − 3.6	< 0.0001
BMI (kg/m^2^)	− 0.1	− 0.1, 0.3	0.18
Sleep (h)	− 0.3	− 0.2, 0.9	0.23
**CNBD:CNFD ratio (min.–max: 0.4–4.1)**			
Schizophrenia	− 0.3	− 0.9, 0.3	0.33
Cingulate gyrus (ICV %)	4.6	1.2, 8.1	0.01
Hippocampus (ICV %)	3.1	− 2.5, 8.6	0.26
ESC feet (µS)	0.02	0.01, 0.03	< 0.01
**Cingulate gyrus (ICV %) (min.–max: 0.70–1.04)**			
Schizophrenia	− 0.07	− 0.13, − 0.01	< 0.05

All the variables considered in the fitted model had P < 0.05.

The cingulate gyrus volume includes Rostral Anterior Cingulate Gyrus and Caudal Anterior Cingulate Gyrus.

*CNFD* corneal nerve fiber density, *CNFL* length, *CNBD* branch density, *VPT* vibration perception threshold, *ESC* electrochemical skin conductance, *ICV* intra cranial volume.

**Figure 1:**
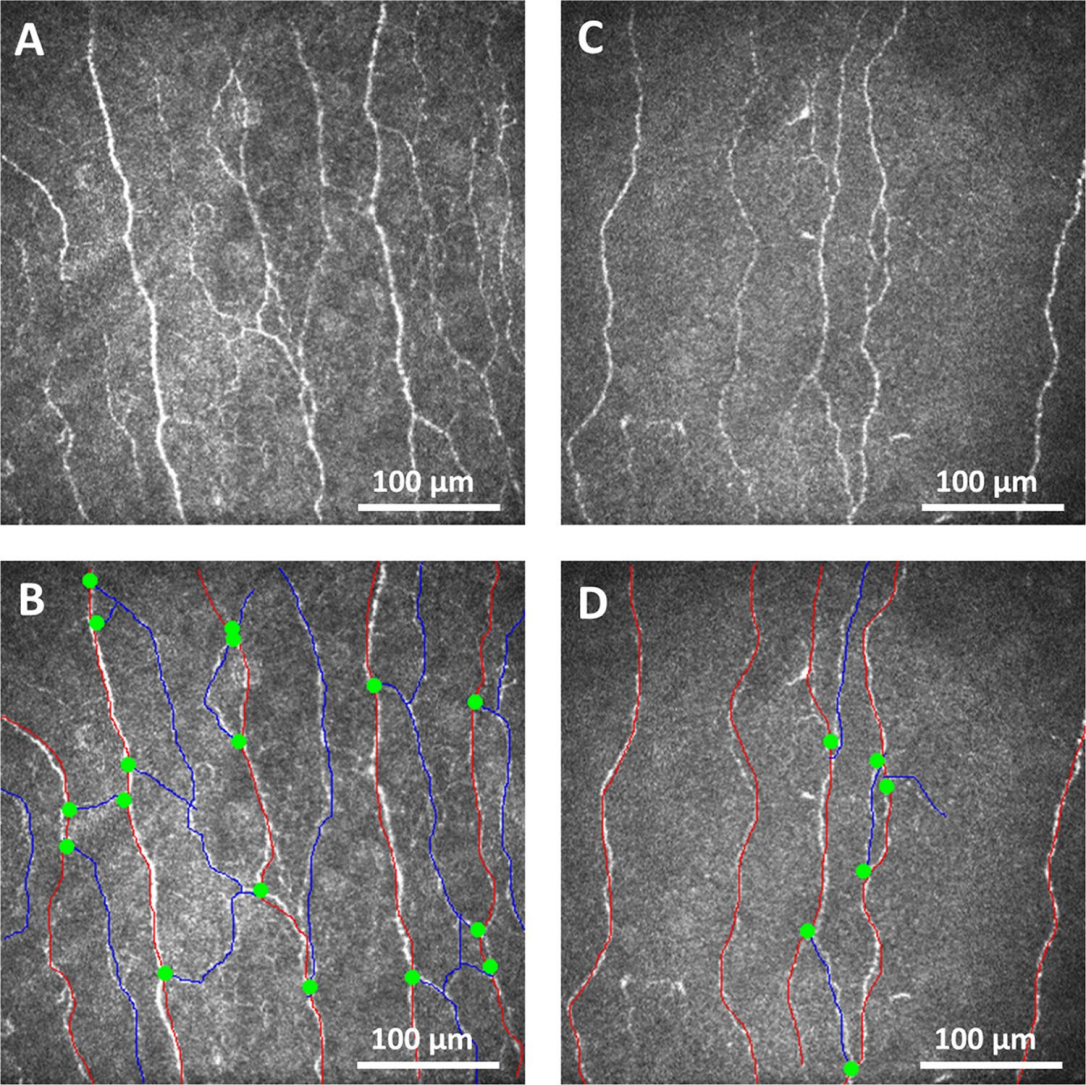
Corneal nerve fiber morphology in a healthy control and subject with schizophrenia. Corneal confocal microscopy (CCM) images of the sub-basal nerve plexus from a health control subject (**A**) with the nerves traced (**B**) and a subject with schizophrenia (**C**) with nerves traced (**D**) showing reduced corneal nerve fiber density (red lines), branch density (green dots) and length (red and blue lines) in the subject with schizophrenia compared to the healthy control.

All subjects completed CCM assessments without expressing any concerns about the eye drop or contact of the cornea with the TomoCap. Subjects with schizophrenia had a significantly lower CNFD (fibers/mm^2^) (27.9 ± 9.1 vs 37.3 ± 6.7, t = 4.6, P < 0.0001), CNBD (branches/mm^2^) (41.9 ± 28.9 vs 87.6 ± 29.3, t = 6.0, P < 0.0001), CNFL (mm/mm^2^) (16.7 ± 5.4 vs 24.6 ± 3.9, t = 6.5, P < 0.0001) and CNBD:CNFD ratio (1.4 ± 0.7 vs 2.4 ± 0.8, t = 4.7, P < 0.0001) compared to controls (Fig. 1 and Table 1). Subjects with schizophrenia without diabetes also had a significantly lower CNFD (fibers/mm^2^) (26.8 ± 7.8 vs 37.3 ± 6.7, t = 5.0, P < 0.0001), CNBD (branches/mm^2^) (40.0 ± 23.5 vs 87.6 ± 29.3, t = 6.3, P < 0.0001), CNFL (mm/mm^2^) (16.2 ± 4.2 vs 24.6 ± 3.9, t = 7.2, P < 0.0001) and CNBD:CNFD ratio (1.5 ± 0.7 vs 2.4 ± 0.8, t = 4.4, P < 0.0001 compared to controls.

As shown in Table 2, CNFD adjusted for cigarette smoking, BMI, and VPT was significantly and negatively associated with schizophrenia (β coefficient: − 5.2 fibers/mm^2^, 95% CI − 9.4, − 1.0, P < 0.05). Reduced CNFD remained significantly and independently associated with cigarette smoking (P < 0.01), BMI (P = 0.01), and VPT (P = 0.01). Similarly, CNBD (β coefficient: − 41.4 branches/mm^2^, 95% CI − 62.0, − 20.8, P < 0.0001) and CNFL (β coefficient − 7.1 mm/mm^2^, 95% CI − 10.6, − 3.6, P < 0.0001) adjusted for BMI and sleep were significantly and negatively associated with schizophrenia. CNBD:CNFD ratio adjusted for cingulate gyrus and hippocampus volumes and sudomotor function lost its significant association with schizophrenia (P = 0.33). In the multiple linear regression analysis, CNBD:CNFD ratio remained significantly and positively associated with cingulate gyrus volume (P = 0.01) and sudomotor function (P < 0.01). After adjusting for the imbalances between subjects with schizophrenia and healthy controls for diabetes, hypertension, hyperlipidemia and BMI, schizophrenia remained a significant independent variable for corneal nerve measures but the r^2^ was lower for CNFD (r^2^: 0.40 vs 0.43), CNBD (r^2^: 0.37 vs 0.41) and CNFL (r^2^: 0.41 vs 0.42).

Corneal nerve measures had no significant association with metabolic syndrome (P = 0.61–0.64), diabetes (P = 0.057–0.54) (Supplementary Table 2), antipsychotic drugs with the highest risk of dry eyes (1–10%) (P = 0.48–0.78), symptom severity of schizophrenia (P = 0.35–0.86), hemoglobin (P = 0.32–0.50), cognitive function (P = 0.051–0.67), antidepressants (P = 0.18–0.92) and treatment for movement disorders (P = 0.28–0.51). Supplementary Table 2 shows that there was a small non-significant loss of corneal nerves in those with (n = 10) compared to without (n = 52) diabetes.

### Volumetric brain MRI (Tables 1 and 2)

Whole brain (P = 0.61), cortical gray matter (P = 0.99), ventricle (P = 0.47), hippocampus (P = 0.10), amygdala (P = 0.68), thalamus (P = 0.20) and entorhinal cortex (P = 0.26) volumes did not differ, but the mean cingulate gyrus volume was significantly lower in subjects with schizophrenia compared to healthy controls (0.83 ± 0.07 vs 0.89 ± 0.08, P < 0.05, Cohen’s d = 0.80).

Cingulate gyrus volume was significantly and negatively associated with schizophrenia (β coefficient: − 0.07, 95% CI − 0.13, − 0.01, P < 0.05). Adjustment for imbalances between subjects with schizophrenia and healthy controls resulted in a sharp drop in the adjusted r^2^ (r^2^: 0.13 vs 0.08) with minimal change for the β coefficient (− 0.08) and thus the earlier model was adopted.

Cingulate gyrus volume showed no association with symptom severity of schizophrenia (P = 0.50), hemoglobin (P = 0.64), cognitive function (P = 0.49), antidepressants (P = 0.85), and treatment for movement disorders (P = 0.89).

### Diagnostic utility for schizophrenia (Table 3 and Fig. 2)

**Table 3:** Diagnostic accuracy of montreal cognitive assessment (MoCA), corneal confocal microscopy (CCM) and cingulate gyrus volume for schizophrenia.

	AUC % (95% Cl)	Cutoff value	Sensitivity %	Specificity %	*P*-value
MoCA score	70 (57–84)	≤ 27	73	58	< 0.01
CNFD (fibers/mm^2^)	79 (68–91)	≤ 36	79	58	< 0.0001
CNBD (branches/mm^2^)	88 (79–97)	≤ 61	82	85	< 0.0001
CNFL (mm/mm^2^)	88 (79–96)	≤ 21	79	81	< 0.0001
CNBD:CNFD ratio	84 (74–95)	≤ 1.9	85	81	< 0.0001
Cingulate gyrus (ICV %)	73 (54–92)	≤ 0.84	61	80	< 0.05

*AUC* area under the curve, *CNFD* corneal nerve fiber density, *CNBD* branch density, *CNFL* fiber length.

**Figure 2:**
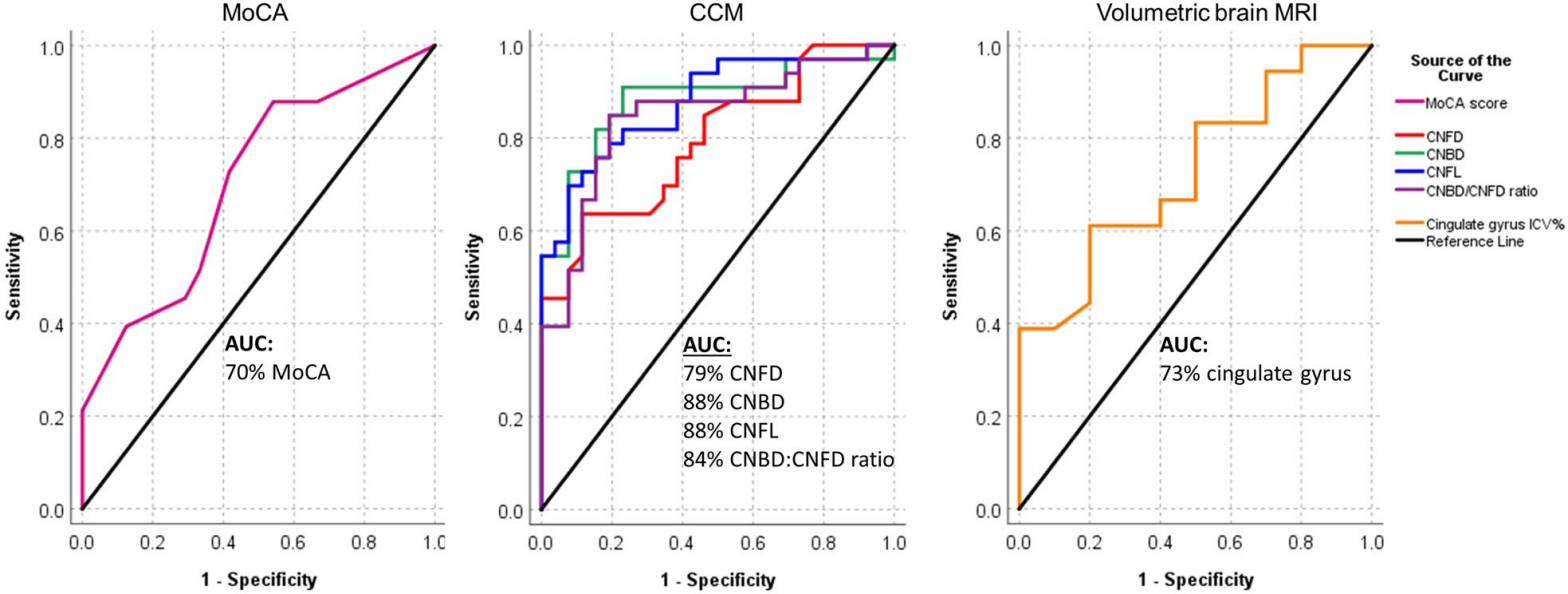
ROC curves showing the diagnostic accuracy of MoCA, corneal nerve fiber measures and cingulate gyrus volume for schizophrenia. ROC analysis expressed as area under the curve (AUC) to determine the effectiveness of MoCA, corneal nerve fiber measures and cingulate gyrus volume to discriminate subjects with schizophrenia from healthy controls.

The AUC to distinguish subjects with schizophrenia from healthy controls was 88% for CNFL (P < 0.0001), 84% for CNBD (P < 0.0001), 84% for CNBD:CNFD ratio (P < 0.0001), 79% for CNFD (P < 0.0001), 73% for the cingulate gyrus ICV% (P < 0.05) and 70% for MoCA (P < 0.01). The sensitivity and specificity for schizophrenia was 82% and 85% with a CNBD cut-off of ≤ 61 branches/mm^2^, 79% and 81% with a CNFL cut-off of ≤ 21 mm/mm^2^, 85% and 81% with a CNBD:CNFD ratio cut-off of ≤ 1.9, 79% and 58% with a CNFD cut-off of ≤ 36 fibers/mm^2^, 61% and 80% with a cingulate gyrus volume cut-off of ≤ 0.84 ICV%, and 73% and 58% with a MoCA cut-off of ≤ 27.

## Discussion

This study shows evidence of reduced corneal nerve fibers and cingulate gyrus volume in patients with schizophrenia. Furthermore, corneal nerve fiber loss in patients with schizophrenia was not associated with diabetes or metabolic syndrome. We cannot differentiate whether the axonal loss is neurodevelopmental or neurodegenerative in origin^1,2^. However our results provide insights into the neuropathology in schizophrenia^27^. A key feature is the reduced corneal nerve branch density and branch to fiber density ratio, which also have the highest diagnostic ability to distinguish patients with schizophrenia from healthy controls, indicating a greater impact on more distal nerves.

Neuroimaging studies have identified a pattern of MRI brain abnormalities related to schizophrenia^28,29^. The ENIGMA consortium^28^ assessed 2028 patients with schizophrenia and reported a reduced volume of hippocampus, amygdala, thalamus and nucleus accumbens and a higher volume of the pallidum and lateral ventricles. Kong et al. reported subtle neurological abnormalities of motor and sensory function and associated them with abnormal organization of structural brain networks in schizophrenia^30^. However, other studies^3^ have reported no difference in the volume of the whole brain, hippocampus, amygdala, caudate and ventricles between patients with schizophrenia and healthy controls. The current study confirms no change in the volume of the hippocampus, amygdala, thalamus, entorhinal cortex, ventricles, cortical gray matter or whole brain, but does show a reduced volume of the cingulate gyrus which has been reported in schizophrenia^29^. These conflicting findings might be attributed to differing cohort size and study populations as well as different software used to quantify the brain volume (NeuroQuant vs Freesurfer), which have low intraclass correlation coefficient for some brain structures. Whilst a lower cingulate gyrus volume correlated with an increased number of perseverative errors on cognitive testing^31^, increased anterior cingulate volume correlated with greater improvement in positive symptoms^32^. Furthermore, longitudinal MRI studies^5,31,32^ have shown a progressive reduction in the volume of the cingulate gyrus, thalamus, frontal, temporal and parietal lobe, and ventricular enlargement in patients with schizophrenia over 1–7 years, particularly 2–3 years after the onset of disease^32^. However, a meta-analysis of 80 studies on brain MRI in schizophrenia found no conclusive evidence to support its diagnostic or prognostic validity^33^. Indeed, structural brain MRI has not been recommended as a diagnostic marker of neuronal pathology in schizophrenia based on the lack of both an established pattern of neuronal pathology^7,33^ and unequivocal association with psychosis^6^.

Studies using CCM have reported corneal neurodegeneration in dementia, multiple sclerosis, Parkinson’s disease and Friedreich’s ataxia^34^. This study found a significant reduction in corneal nerve fiber density, length, and branch density as well as branch to fiber density ratio in patients with schizophrenia. A postmortem study has shown that patients with schizophrenia have a comparable pyramidal neuron density but reduced branching and cell size in layer 3 of the primary visual cortex compared to healthy controls^35^. Impaired neuronal branching^35,36^ might have a negative impact on the transition of the neuronal network from a child- to an adult-brain and the development of higher-order cognition^37^. This study also shows that corneal nerve branch to fiber density ratio correlated positively with the volume of the cingulate gyrus after adjusting for confounders.

This study shows that CCM had a higher accuracy than cingulate gyrus volume and cognitive function for distinguishing patients with schizophrenia from healthy controls with a sensitivity and specificity exceeding 80%. Studies that have assessed the retina of patients with schizophrenia utilizing electroretinography (ERG) and optical coherence tomography (OCT) have also shown a reduction in retinal nerve fiber layer and macular volume and a reduction in photoreceptor and bipolar cell activity compared to controls^8^. It is possible that dopamine dysregulation^9^, systemic inflammation and hypoxia^38^ may affect not only the brain but also the retina and cornea.

The association between neuronal pathology and symptom severity in schizophrenia has a conflicting literature. Indeed, we show that corneal nerve fiber measures and cingulate gyrus volume were not associated with symptom severity or cognitive function, although, this may reflect the effect of ongoing treatment. Nevertheless, some studies have shown that a reduction of gray matter volume, including the cingulate gyrus volume was associated with worsening^32^ or less improvement^39^ of symptoms, whereas other studies have found no association^4^.

Some patients with schizophrenia experience distorted colour or brightness, hallucinations, or altered contrast sensitivity, which has been attributed to cortical, subcortical and retinal dysfunction^40^, but our findings also suggest that the loss of corneal nerves may impact on ocular surface health through reduced tearing and blinking. Corneal nerves also release various trophic factors, which maintain epithelial integrity^41^. Diabetes and obesity are associated with corneal nerve fiber loss and the severity of peripheral neuropathy^34^. In this cohort of patients with schizophrenia, there was a small non-significant loss of corneal nerves in those with diabetes, but no association with metabolic syndrome. This may be attributed to the excellent glycemic control in patients with diabetes (HbA1c 6.6%). Although diabetes is associated with corneal nerve loss^14^, this study shows that the loss of corneal nerve fibers in patients with schizophrenia remained significant after excluding those with diabetes. Indeed, there was no abnormality in vibration perception or sudomotor function, indicating no evidence of peripheral neuropathy in these patients.

We acknowledge the small sample size is a limitation of the current study and hence, the diagnostic accuracy of CCM and MRI brain volume for schizophrenia should be interpreted with caution. We have also not assessed for severe dry eye, which can be caused by antipsychotic drug use and is associated with corneal nerve abnormalities^41^. However, this study shows that corneal nerve measures had no association with antipsychotic drugs with the highest risk of dry eyes, antidepressants or treatment for movement disorders. Nevertheless, this is the first study to show a loss of corneal nerves in patients with schizophrenia and warrants a larger longitudinal cohort study to (1) confirm the current findings, (2) assess whether the disproportionately lower nerve branch to fiber density ratio is a distinctive neuropathological characteristic of schizophrenia, and (3) establish the predictive ability of CCM for worsening schizophrenia or relapses.

## Supplementary Information

Supplementary Table 1.

Supplementary Table 2.

## Data Availability

The data that support the findings of this study are available on request from the corresponding author.
