# Supplementary material for: Abnormal corneal nerve morphology and brain volume in patients with schizophrenia
Source: Sci Rep. 2022 Feb 3;12:1870. doi: 10.1038/s41598-022-05609-w (PMC8814184; doi:10.1038/s41598-022-05609-w)
Supplement: Supplementary file 1 — Supplementary Table 1. [file 41598_2022_5609_MOESM1_ESM.docx]

**Supplementary Table 1.** Comparison of clinical and metabolic characteristics between subjects with schizophrenia and control subjects.

|  | Controls  (n=26) | Schizophrenia  (n=36) | P value | Phi/ Cohen’s d |
| --- | --- | --- | --- | --- |
| Age, years | 33.7±11.1 | 35.3±11.1 | 0.59 |  |
| Male, n (%) | 16/26 (61.5) | 26/36 (72.2) | 0.38 |  |
| Hypertension, n (%) | 3/26 (11.5) | 16/36 (44.4) | <0.01 | 0.35 |
| Systolic BP, mmHg | 120.7±11.7 | 120.6±14.1 | 0.98 |  |
| Diastolic BP, mmHg | 75.1±11.3 | 73.7±8.6 | 0.59 |  |
| Obesity, n (%) | 8/23 (34.8) | 18/35 (51.4) | 0.21 |  |
| BMI, kg/m^2^ | 27.5±5.2 | 32.9±9.1 | <0.01 | 0.73 |
| Diabetes, n (%) | 0/26 (0) | 10/36 (27.8) | <0.001 |  |
| HbA1c, mmol/mol | 35.3±3.8 | 39.5±11.9 | 0.06 |  |
| HbA1c, % | 5.4±0.3 | 5.8±1.1 |  |  |
| Hyperlipidemia, n (%) | 2/26 (7.7) | 12/36 (33.3) | <0.05 | 0.30 |
| Total cholesterol, mmol/l | 4.8±0.8 | 4.5±1.1 | 0.32 |  |
| Triglyceride, mmol/l | 1.7±1.8 | 1.8±0.9 | 0.88 |  |
| HDL, mmol/l | 1.2±0.3 | 1.2±0.3 | 0.64 |  |
| LDL, mmol/l | 2.9±0.7 | 2.7±0.9 | 0.39 |  |
| Creatinine, µmol/L | 67.5±22.9 | 65.8±18.7 | 0.80 |  |
| Vitamin B_12_, pmol/L | 436.9±475.3 | 321.4±157.3 | 0.39 |  |
| Vitamin D, ng/ml | 18.9±11.1 | 16.6±7.8 | 0.47 |  |
| Smoking cigarettes, n (%) | 4/26 (15.4) | 10/36 (27.8) | 0.25 |  |

Numeric variables and frequency distribution for categorical variables are summarized as means ± standard deviation or n (%). Variables were compared using unpaired t-test. Categorical variables were compared using x^2^. Abbreviations: blood pressure (BP).
