# Supplementary material for: Abnormal corneal nerve morphology and brain volume in patients with schizophrenia
Source: Sci Rep. 2022 Feb 3;12:1870. doi: 10.1038/s41598-022-05609-w (PMC8814184; doi:10.1038/s41598-022-05609-w)
Supplement: Supplementary file 2 — Supplementary Table 2. [file 41598_2022_5609_MOESM2_ESM.docx]

**Supplementary Table 2.** Comparison of HbA1c and corneal nerve fiber measures between subjects with and without diabetes.

|  | No diabetes  (n=52) | Diabetes  (n=10) | P value |
| --- | --- | --- | --- |
| HbA1c, % | 5.4±0.4 | 6.6±1.6 | <0.05 |
| CNFD, fibers/mm^2^ | 32.4±8.9 | 30.4±11.5 | 0.61 |
| CNBD, branches/mm^2^ | 65.2±35.7 | 46.3±40.0 | 0.19 |
| CNFL, mm/mm^2^ | 20.7±5.8 | 17.8±7.6 | 0.28 |
| CNBD:CNFD ratio | 1.9±0.8 | 1.4±0.9 | 0.09 |

Variables are summarized as means ± standard deviation. Variables were compared using unpaired t-test. Abbreviations: corneal nerve fiber density (CNFD), length (CNFL), branch density (CNBD).
